# From run-to-failure to condition-based care: a multi-domain framework for preventive psychiatry

**DOI:** 10.3389/fpsyt.2026.1821471

**Published:** 2026-04-22

**Authors:** Samir El Alaoui

**Affiliations:** Centre for Psychiatry Research, Department of Clinical Neuroscience, Karolinska Institutet & Stockholm Health Care Services, Region Stockholm, Stockholm, Sweden

**Keywords:** allostatic load, clinical staging, condition-based care, glymphatic system, nutritional psychiatry, preventive psychiatry, psychoneuroimmunology, subclinical intervention

## Abstract

Mental disorders are the leading cause of years lived with disability worldwide, yet the dominant clinical model remains reactive, intervening only after diagnostic thresholds are met. Drawing on the engineering distinction between run-to-failure and condition-based maintenance, this review argues that psychiatry faces a quantifiable intervention threshold gap. Synthesising recent meta-analytic evidence, we show that psychological interventions at subclinical symptom levels reduce major depression incidence by 43% at post-treatment and 33% at 12-month follow-up (individual-participant-data meta-analysis; 30 trials; *N* = 7,201), that shorter duration of untreated illness is associated with 70% greater likelihood of treatment response, and that early intervention for first-episode psychosis reduces hospitalisation by 26%. We review convergent evidence across five modifiable domains — sleep and glymphatic clearance, nutritional psychiatry, allostatic load regulation, autonomic function, and psychoneuroimmunological monitoring — selected for objective measurability, meta-analytic interventional support, identified mechanistic pathways, and population-level scalability. These domains form a mechanistically interconnected network in which deterioration in one can cascade across others. Clinical staging models, adapted from oncology, provide the graduated diagnostic architecture for condition-based care. We present a speculative multi-domain monitoring protocol with parameters, frequencies, and action thresholds calibrated to clinical stage. Among the five domains, inflammatory monitoring via C-reactive protein emerges as the most implementation-ready, with established clinical thresholds, demonstrated treatment-selection utility, and symptom-specific associations with neurovegetative features consistent with an immuno-metabolic depression subtype. Prospective multi-domain monitoring trials are identified as the most urgent research priority.

## 1 Introduction

Mental disorders are now the leading cause of years lived with disability worldwide, with a large-scale meta-analysis of 192 epidemiological studies establishing that the peak age of onset varies from 5.5 years for anxiety disorders to 20.5 years for mood disorders, and that 62.5% of all mental disorders have their onset before age 25 (1). Despite decades of accumulating evidence that these disorders follow progressive, stage-based trajectories amenable to early intervention (2, 3), the dominant clinical model remains fundamentally reactive. Patients are typically permitted to reach established diagnostic thresholds—and frequently to progress well beyond them—before meaningful treatment is initiated. World Health Organization data from over 76,000 respondents across 15 countries document median treatment delays of 3–30 years for anxiety disorders and 1–14 years for mood disorders from first symptom onset (4).

This pattern mirrors what maintenance engineering terms a *run-to-failure* strategy: permitting a system to degrade until catastrophic breakdown necessitates emergency repair (5). In industrial contexts, the inefficiency and costliness of this approach led to its systematic replacement by condition-based maintenance—a paradigm in which continuous monitoring of key system parameters triggers intervention at the earliest signs of degradation, well before functional failure occurs (5). The conceptual parallel with psychiatric care is striking, and was first articulated in a nursing concept analysis that transposed condition-based maintenance to chronic illness management (6). The present review extends this analogy to psychiatry specifically, proposing that clinical staging models can provide the diagnostic architecture needed to operationalise condition-based psychiatric care.

We synthesise recent high-quality meta-analytic evidence to argue that psychiatry faces a quantifiable *intervention threshold gap*: a measurable discrepancy between the point at which current guidelines recommend intervention and the point at which the evidence shows intervention is maximally effective. We organise the supporting evidence around five domains of modifiable biological and behavioural risk — sleep, nutrition, stress physiology, autonomic function, and immune signalling — selected for their objective measurability, meta-analytic interventional support, identified mechanistic pathways, and population-level scalability. We propose that clinical staging models (7, 8) provide the graduated diagnostic architecture needed to operationalise condition-based care across these domains.

## 2 The intervention threshold gap

Current clinical guidelines, including the US Preventive Services Task Force recommendation for universal adult depression screening, endorse population-level screening but define the intervention threshold at the point of established diagnosis and do not specify an optimal screening frequency (9). This approach establishes a clinical floor rather than an evidence-based optimum.

The most definitive challenge to this threshold comes from Buntrock et al.’s (10) individual-participant-data meta-analysis — the highest level of meta-analytic evidence, in which raw data from all included trials are reanalysed in a single statistical framework — encompassing 30 randomised controlled trials and 7,201 adults with subthreshold depressive symptoms.

Psychological interventions delivered before diagnostic criteria were met reduced the incidence of major depressive disorder (MDD) by 43% at post-treatment (incidence rate ratio [IRR] = 0.57, 95% CI: 0.35–0.93), 42% within six months (IRR = 0.58, 95% CI: 0.39–0.88), and 33% at 12 months (IRR = 0.67, 95% CI: 0.51–0.88). These effects were not sustained at 24 months, suggesting that, like any maintenance protocol, periodic re-application is necessary. Moderator analyses indicated that preventive effects were most pronounced among individuals with moderately severe baseline symptoms and those without prior psychotherapy exposure—findings with direct implications for resource allocation in stepped-care models.

The costs of exceeding the optimal intervention window are well documented. A systematic review of duration of untreated illness in depression found that shorter time from symptom onset to treatment initiation was associated with 70% greater likelihood of treatment response (RR = 1.70) and 65% greater likelihood of remission (RR = 1.65) (11). In psychosis, early intervention services for first-episode patients reduce hospitalisation by 26% (RR = 0.74) and treatment discontinuation by 30% (RR = 0.70) compared to treatment-as-usual (12). That substantial gains are achievable even at the point of initial diagnosis (corresponding to Stage 2 in staging nomenclature) underscores the likely magnitude of benefits attainable through intervention at earlier, pre-threshold stages — a hypothesis now directly supported by the Buntrock et al. (2024) findings for depression (10).

The question, then, is how to identify individuals who have entered the intervention window but have not yet crossed the diagnostic threshold. Clinical staging models — adapted from oncology, where staging guides treatment intensity to disease progression — provide a graduated framework for doing so. Rather than treating mental illness as either absent or present, staging classifies individuals along a continuum from asymptomatic but at-risk states through early symptomatic presentations to established and treatment-resistant disorder, enabling clinicians to match intervention intensity to illness severity. The transdiagnostic consensus model (8), refined in the Staging 2.0 proposal (7), operationalises each stage across three dimensions — symptom severity, functional capacity, and neurocognitive performance — defining progression from asymptomatic at-risk states (Stage 0), through non-specific symptoms with mild functional change (Stage 1a), attenuated but sub-threshold syndromes with measurable functional decline (Stage 1b), to full-threshold disorder (Stage 2) and severe, treatment-resistant illness (Stages 3–4). These staging models function as the psychiatric equivalent of condition-monitoring systems: they detect degradation before it consolidates into disorder — but only if clinicians are actively monitoring. Importantly, staging does not require continuous reassessment; rather, it provides a framework for interpreting monitoring data collected at stage-appropriate intervals, with longer intervals at Stage 0 and progressively shorter intervals as subclinical signs emerge, analogous to the adaptive inspection schedules used in condition-based maintenance (see **Table 1** for a proposed protocol).

**Table 1:** Proposed multi-domain condition-based monitoring protocol (speculative).

Domain	Parameter(s)	Measurement	Frequency	Clinical action threshold
Sleep	Sleep duration, efficiency, slow-wave sleep proportion	Actigraphy (research-grade or consumer-grade accelerometry); polysomnography for clinical confirmation	Continuous (wearable) with monthly review	Sustained sleep efficiency <85% or slow-wave sleep reduction >1 SD from individual baseline over ≥2 weeks
Nutrition	Dietary quality (e.g., Mediterranean Diet Score); gut inflammatory markers	Validated food frequency questionnaire; faecal calprotectin	Quarterly (questionnaire); 6-monthly (biomarker)	MDS decline ≥2 points from baseline; elevated faecal calprotectin
Allostatic load	Composite index: cortisol, blood pressure, waist-hip ratio, HbA1c, lipid panel	Routine blood panel + anthropometrics	6-monthly (Stage 0); quarterly (Stage 1a+)	AL index increase ≥1 component above individual baseline
Autonomic function	Resting heart rate variability (RMSSD); respiratory rate	Ambulatory HRV recording (research-grade or consumer-grade devices)	Continuous (wearable) with monthly review	RMSSD decline >1 SD sustained over ≥2 weeks
Immune	CRP; IL-6 for high-risk individuals	Standard blood panel	6-monthly (Stage 0); quarterly (Stage 1a+)	CRP >3 mg/L = low-grade inflammation (present in ~27% of depressed patients); CRP 1–3 mg/L = elevated risk; CRP <1 mg/L = low inflammatory risk. Thresholds may additionally inform treatment selection: CRP ≥1 mg/L predicts preferential response to noradrenergic over serotonergic antidepressants (42). Elevated CRP in the context of neurovegetative symptoms (fatigue, hypersomnia, increased appetite) may represent a stronger clinical signal than isolated CRP elevation.

This protocol is speculative and is presented to illustrate the conceptual feasibility of multi-domain condition-based monitoring, not as validated clinical guidance. Monitoring frequencies are calibrated to two factors: the temporal dynamics of each biological parameter (sleep and autonomic function fluctuate over days–weeks; inflammatory and allostatic markers over months) and the individual’s clinical stage (intervals shorten from 6-monthly at Stage 0 to quarterly at Stage 1a+). Action thresholds are defined relative to individual baselines where feasible, consistent with the condition-based maintenance principle that deterioration from a known state is more informative than deviation from a population mean. CRP thresholds follow established cardiovascular risk categories (<1 mg/L = low, 1–3 mg/L = medium, >3 mg/L = high; Pearson et al., 2003) 48 and have demonstrated treatment-selection utility in depression (Uher et al., 2014) (42). Elevated CRP in the context of energy-related neurovegetative symptoms — fatigue, hypersomnia, increased appetite — may represent a stronger signal for intervention than isolated CRP elevation, consistent with the immuno-metabolic depression subtype (Milaneschi et al., (40); Milaneschi et al., (39)). None of the proposed multi-domain thresholds or monitoring intervals have been validated in prospective trials; their empirical testing represents a priority for future research. AL, allostatic load; CRP, C-reactive protein; HbA1c, glycated haemoglobin; HRV, heart rate variability; IL-6, interleukin-6; MDS, Mediterranean Diet Score; RMSSD, root mean square of successive differences; SD, standard deviation.

## 3 Multi-domain evidence for condition-based care

A condition-based approach to psychiatric care must extend beyond symptom monitoring to encompass the biological substrates that underlie vulnerability and resilience. From a larger set of modifiable risk factors with established psychiatric associations — including physical activity, social connectedness, substance use, and environmental exposures — we selected five domains for detailed review: sleep and glymphatic clearance, nutritional psychiatry, allostatic load regulation, autonomic function, and psychoneuroimmunological monitoring. The selection was guided by criteria consistent with the condition-based maintenance analogy: each domain involves an objectively measurable biological parameter amenable to longitudinal monitoring, has demonstrated interventional efficacy at the meta-analytic level, operates through identified mechanistic pathways, and is modifiable at a cost and scale compatible with population-level implementation.

**Figure 1:**
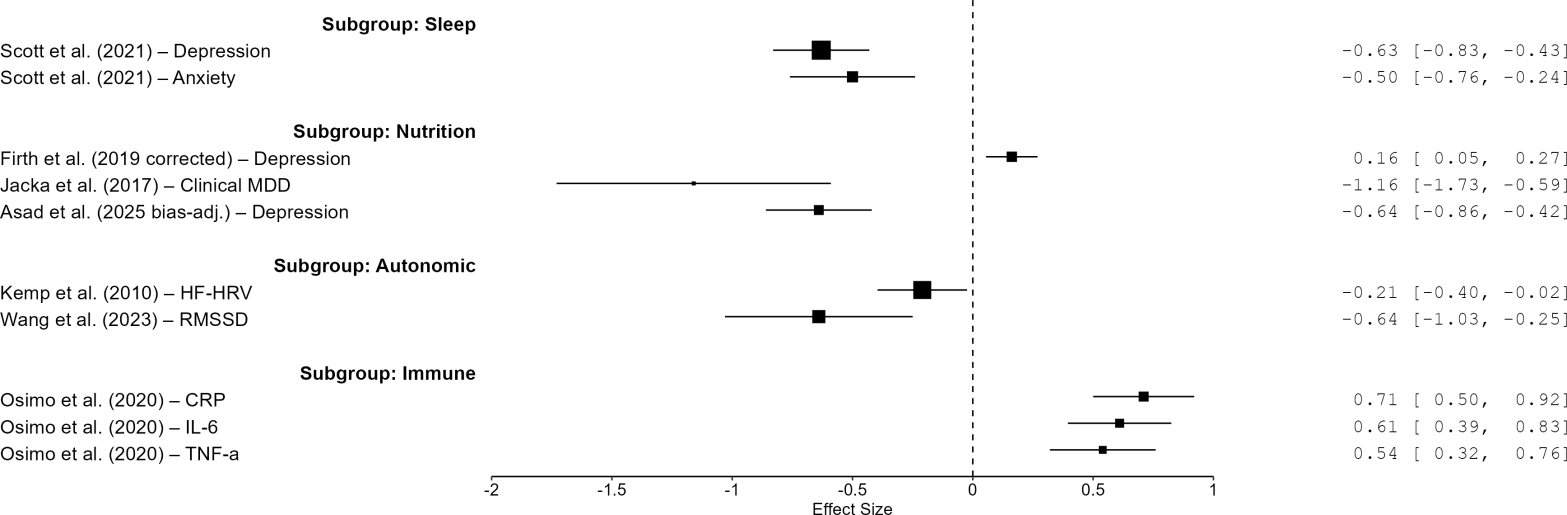
Meta-analytic evidence for modifiable domains in preventive psychiatry. This forest plot summarises key meta-analytic findings across the four domains for which standardised effect sizes are available. In the Sleep domain, effect sizes reflect how much improving sleep quality reduces symptoms of depression and anxiety. In the Nutrition domain, effect sizes reflect the impact of dietary improvement and probiotic supplementation on depressive symptoms. In the Autonomic domain, effect sizes reflect reduced heart rate variability in patients with depression compared with healthy controls. In the Immune domain, effect sizes reflect elevated inflammatory markers in patients with depression compared with healthy controls. All values are Hedges’ g, Cohen’s d, or standardised mean differences (SMD) with 95% confidence intervals. Larger squares indicate more precise estimates. Sources vary in design and directionality; cross-domain comparison should be interpreted with caution (see Section 3). One additional domain and the core prevention finding use ratio-scale metrics not shown in this plot. For allostatic load, Gou et al. (2025) (25) found that individuals with high cumulative physiological stress had a 39% increased risk of depression (HR = 1.39, 95% CI: 1.31–1.48), 30% increased risk of anxiety (HR = 1.30, 1.23–1.38), and 43% increased risk of suicide (HR = 1.43, 1.12–1.84) over 13 years of follow-up. For the core finding, Buntrock et al. (2024) (10) found that psychological interventions delivered before diagnostic criteria were met reduced the incidence of major depression by 43% (IRR = 0.57, 95% CI: 0.35–0.93).

These domains are not independent. They interact through shared biological pathways — inflammation modulates sleep architecture and gut permeability; chronic stress elevates allostatic load while suppressing vagal tone; sleep deprivation impairs glymphatic clearance and amplifies inflammatory signalling — forming an interconnected network of vulnerability in which deterioration in one domain can cascade across others. This mechanistic interconnectedness provides the rationale for multi-domain rather than single-domain monitoring: it is the convergence of signals across domains, rather than any individual parameter, that most reliably indicates progression along the clinical staging continuum. We note that the five domains vary in clinical readiness, from inflammatory monitoring via CRP — which has established thresholds and demonstrated treatment-selection utility — to allostatic load indices, which lack a standardised measurement panel (see **Table 2** for a domain-by-domain readiness classification). **Figure 1** presents the key effect sizes from the meta-analytic sources reviewed across four of the five domains (note that effect sizes derive from different metrics and should not be compared directly across domains).

**Table 2:** The intervention threshold gap: current practice versus emerging evidence across key domains of preventive psychiatric care.

Domain	Current practice	Emerging evidence	Clinical readiness	Key sources
Intervention threshold	Diagnostic criteria met (e.g., PHQ-9 ≥ 10; DSM-5 threshold)	Subclinical distress (Stage 1a/1b); 43% MDD incidence reduction at post-treatment with pre-threshold intervention	Emerging: staging instruments under development; no validated clinical decision tool	Buntrock et al. (10); Shah et al. (8); Scott et al. (7)
Screening frequency	Universal screening recommended; no evidence-based interval specified	Stage-adaptive intervals: 6-monthly at Stage 0, quarterly at Stage 1a+ (see **Table 1**)	Research-stage: no evidence-based screening interval established; potential harms of frequent screening incompletely characterised	Barry et al. (9); Lang et al. (44); Fusar-Poli et al. (2)
Sleep & glymphatic clearance	General sleep hygiene advice, typically post hoc	Proactive slow-wave sleep optimisation; single-night deprivation impairs glymphatic clearance; sleep improvement reduces depression (*g* = −0.63)	Emerging: actigraphy and consumer wearables increasingly accessible; polysomnography available but resource-intensive	Iliff et al. (13); Xie et al. (14); Eide et al. (15); Hauglund et al. (16); Scott et al. (17)
Nutritional psychiatry	Rarely integrated into psychiatric care plans	Mediterranean diet as first-line adjunct (NNT = 4.1); probiotics for clinical symptoms (bias-adjusted SMD = −0.64)	Available: validated dietary questionnaires; Emerging: faecal biomarkers for gut inflammation	Jacka et al. (18); Firth et al. (21); Asad et al. (23)
Allostatic load	Addressed reactively after burnout or crisis	Allostatic load biomarker panels as routine monitoring; elevated AL associated with depression (HR = 1.39), anxiety, and suicide	Research-stage: individual components routinely collected but no standardised composite panel, scoring algorithm, or clinical workflow	Gou et al. (25); McEwen & Stellar (24); Goldstein & Kopin (26); Chmiel & Kurpas (27)
Autonomic function	Not routinely assessed in psychiatric evaluations	HRV reduced across psychiatric disorders (RMSSD SMD = −0.64); HRV biofeedback reduces depressive symptoms (*g* = 0.38); ambulatory monitoring feasible via consumer devices	Emerging: consumer-grade ambulatory HRV accessible; evidence strength weak-to-suggestive for depression specifically	Kemp et al. (28); Wang et al. (29); Wang et al. (30); Pizzoli et al. (31); Laborde et al. (32)
Inflammatory markers	Not routinely assessed in psychiatric evaluations	CRP, IL-6, TNF-α as transdiagnostic early-warning indicators; CRP ≥1 mg/L informs antidepressant selection; CRP most strongly associated with neurovegetative symptoms consistent with immuno-metabolic depression subtype	Available: CRP via routine blood panel with established thresholds; IL-6 available but not routinely ordered in psychiatric settings	Osimo et al. (36); Osimo et al. (37); Miller & Raison (38); Uher et al. (42); Milaneschi et al. (39); Milaneschi et al. (40)

This table summarises the discrepancy between current clinical practice and emerging evidence across seven domains relevant to preventive psychiatric care. The “Clinical readiness” column classifies each domain using a three-tier system: Available indicates that the relevant parameter can be assessed using existing clinical infrastructure and validated instruments; Emerging indicates that measurement tools exist but are not yet routinely integrated into psychiatric care or lack standardised implementation protocols; Research-stage indicates that either no standardised measurement approach exists or that the evidence base is insufficient to support routine clinical application. Clinical readiness varies substantially across domains: inflammatory monitoring via CRP is the most implementation-ready parameter, with established clinical thresholds (<1, 1–3, >3 mg/L; Pearson et al., 2003) (48), demonstrated treatment-selection utility (Uher et al., (42)), symptom-specific associations with neurovegetative features consistent with the immuno-metabolic depression subtype (Milaneschi et al., (39); Milaneschi et al., (40)), and routine availability through standard blood panels. By contrast, allostatic load monitoring remains the least mature, lacking a consensus biomarker panel or validated scoring algorithm. Autonomic function monitoring via HRV occupies an intermediate position: consumer-grade ambulatory measurement is feasible, but the evidence base for depression specifically has been classified as weak-to-suggestive in an umbrella review (Wang et al., (30)). Dietary assessment via validated questionnaires (e.g., Mediterranean Diet Score) is immediately feasible, while gut biomarkers (e.g., faecal calprotectin) remain emerging. Sleep monitoring is increasingly accessible through consumer-grade actigraphy, though polysomnographic confirmation remains resource-intensive. The probiotics effect size reported in the Emerging Evidence column reflects the bias-adjusted estimate (SMD = −0.64) after exclusion of studies with high risk of bias (primary estimate: SMD = −0.96; I² = 85%). AL, allostatic load; CRP, C-reactive protein; DSM-5, Diagnostic and Statistical Manual of Mental Disorders, Fifth Edition; HR, hazard ratio; HRV, heart rate variability; IL-6, interleukin-6; MDD, major depressive disorder; NNT, number needed to treat; PHQ-9, Patient Health Questionnaire-9; RMSSD, root mean square of successive differences; SMD, standardised mean difference; TNF-α, tumour necrosis factor alpha.

### 3.1 Sleep and glymphatic clearance

During wakefulness, metabolic activity generates neurotoxic waste products — including amyloid-β and tau, proteins implicated in neurodegeneration — that accumulate in the spaces between brain cells. The glymphatic system — a perivascular clearance network first described by Iliff et al. (2012) (13) — clears these waste products by driving cerebrospinal fluid along channels surrounding the brain’s blood vessels, effectively flushing the brain’s interstitial spaces. This process depends on aquaporin-4 water channels on astrocyte endfeet and occurs predominantly during deep non-rapid-eye-movement (NREM) sleep, when the interstitial space expands by approximately 60%, dramatically increasing the efficiency of waste clearance (14). Critically, a single night of total sleep deprivation measurably impairs this clearance in humans, and the deficit is not fully compensated by recovery sleep (15) — suggesting that the consequences of sleep disruption are not merely subjective but involve a quantifiable failure of a basic neuroprotective mechanism.

The proximate driver of this clearance process has recently been identified. Hauglund et al. (2025) (16) demonstrated that slow, rhythmic pulsations in blood vessel diameter — controlled by oscillations in norepinephrine released from the locus coeruleus during NREM sleep — act as a pump, propelling cerebrospinal fluid through the brain. The frequency of these norepinephrine oscillations predicted glymphatic clearance efficiency, providing a direct mechanistic link between sleep microarchitecture and brain waste removal. Of particular clinical concern, the commonly prescribed sleep aid zolpidem was found to suppress these norepinephrine oscillations and reduce glymphatic flow. Given that sedative-hypnotics are among the most widely prescribed medications for insomnia, this finding raises the possibility that pharmacological sleep induction may paradoxically impair the very restorative process that makes sleep beneficial — a hypothesis that warrants urgent investigation in clinical populations.

The psychiatric relevance of sleep disturbance extends beyond glymphatic mechanisms. A meta-analysis of 65 randomised controlled trials (*N* = 8,608) demonstrated that interventions improving sleep quality — predominantly cognitive-behavioural therapy for insomnia (CBT-I) — significantly reduce symptoms of depression (*g* = −0.63), anxiety (*g* = −0.51), and psychological stress (*g* = −0.42) (17). These effect sizes are among the largest reported for any single-domain intervention in this review. Whether the psychiatric benefits of improved sleep are mediated specifically through enhanced glymphatic clearance, or through other pathways including circadian rhythm stabilisation, HPA axis re-regulation, and emotional memory processing, remains an open empirical question — but the magnitude and consistency of the interventional evidence establish sleep as a non-negotiable monitoring parameter in any condition-based preventive framework. Sleep architecture (duration, efficiency, slow-wave sleep proportion) is readily measurable through actigraphy and consumer-grade accelerometry, making it one of the most feasible domains for continuous ambulatory monitoring.

### 3.2 Nutritional psychiatry

The SMILES trial — the first randomised controlled trial of dietary modification for clinical depression — demonstrated that a 12-week Mediterranean diet intervention produced a large effect size (Cohen’s *d* = 1.16, NNT = 4.1, 95% CI: 2.3–27.8) (18). As an unblinded trial with a social-support control condition and modest sample size (N = 67), the SMILES result requires contextualisation: the large effect size may partly reflect non-specific elements of the intervention. Nevertheless, the finding has been conceptually replicated in subsequent trials across diverse populations, including young males (19) and individuals with existing depression supplemented with fish oil (20). A meta-analysis of 16 randomised controlled trials (n = 45,826) confirmed that dietary improvement reduces depressive symptoms, though the pooled effect across predominantly non-clinical samples was modest (*g* = 0.162, 95% CI: 0.055–0.269, *p* = .003) (21). No significant effect was observed for anxiety. The discrepancy between the large individual-trial effect in clinical depression and the smaller meta-analytic estimate reflects the dilution inherent in pooling across heterogeneous, predominantly non-clinical populations — suggesting that dietary intervention may be most potent at the moderate-to-severe end of the symptom spectrum, consistent with a staged approach in which intervention intensity is matched to illness severity.

The gut microbiome constitutes a critical mediating pathway between diet and mental health. When the balance of gut bacteria is disrupted — a state termed dysbiosis — the intestinal lining becomes more permeable, allowing bacterial toxins to leak into the bloodstream. This triggers a systemic inflammatory response that ultimately reaches the brain, where it disrupts the production of neurotransmitters such as serotonin and dopamine that regulate mood (22). A meta-analysis of 23 randomised controlled trials in clinically diagnosed populations found that probiotics reduce depression symptoms (SMD = −0.96, 95% CI: −1.31 to −0.61) (23), though this estimate should be interpreted cautiously: heterogeneity was high (I² = 85%), and Egger’s regression test indicated significant publication bias (*p* <.001). When studies with high risk of bias were excluded, the effect was attenuated by approximately one-third but remained clinically meaningful (SMD = −0.64, 95% CI: −0.86 to −0.42; I² = 44%). A similar pattern was observed for anxiety symptoms (primary SMD = −0.59; reduced to −0.39 after exclusion of high-risk studies). These findings suggest that while the magnitude of the probiotic effect may be smaller than the headline estimate indicates, the direction and significance of the effect are robust to quality-based sensitivity analysis.

Taken together, the dietary and probiotic evidence positions nutrition not as an ancillary lifestyle recommendation but as an intervention domain with effect sizes that, even at conservative estimates, fall within the range of established treatments. For condition-based monitoring, dietary quality is assessable through validated instruments such as the Mediterranean Diet Score, while emerging gut inflammatory biomarkers (e.g., faecal calprotectin) may eventually provide objective biological indicators of microbiome-mediated risk — though the latter remain at an early stage of validation for psychiatric applications.

### 3.3 Allostatic load regulation

The concept of allostatic load — introduced by McEwen and Stellar (1993) (24) to describe the cumulative physiological wear from chronic activation of stress-response systems — captures something that subjective stress measures cannot: the progressive, non-linear biological toll of sustained adversity, quantified through a composite of neuroendocrine, cardiovascular, metabolic, and immunological markers. Unlike acute stress, which is adaptive and self-resolving, allostatic overload represents a state in which the regulatory systems themselves become damaged by the demands placed upon them — a process that unfolds across months to years and that, critically, may progress silently before manifesting as psychiatric symptoms.

Epidemiological evidence supports the psychiatric relevance of this construct. A UK Biobank prospective cohort study of 333,017 adults with a median 13-year follow-up demonstrated that elevated allostatic load indices were positively associated with incident depression (HR = 1.39), anxiety (HR = 1.30), and suicide (HR = 1.43) (25). As an observational study, these associations do not establish causality — unmeasured confounders and reverse causation cannot be excluded — but the prospective design, large sample, and long follow-up strengthen the inference that allostatic load accumulation precedes rather than merely accompanies psychiatric disorder.

At the cellular level, Goldstein and Kopin (2018) (26) have proposed a mechanistic pathway by which sustained catecholaminergic activation could contribute to neurodegenerative pathology. In their model, prolonged stress-related demands on catecholamine-producing neurons lead to vesicular leakage of adrenaline and noradrenaline, which are then metabolised into DOPAL, a toxic aldehyde that promotes misfolding of alpha-synuclein — a protein implicated in Parkinson’s disease and related conditions. While this model is based primarily on intraneuronal biochemistry rather than epidemiological evidence linking psychological stress to neurodegeneration in general populations, it illustrates a plausible molecular mechanism by which the cumulative physiological burden captured by allostatic load indices could, if sustained, have consequences extending beyond psychiatric symptomatology.

At the structural level, neuroimaging studies of burnout reveal measurable brain changes: enlargement of the amygdala (the brain’s threat-detection centre), reduction in prefrontal grey matter (involved in executive control and emotional regulation), and weakened connectivity between these two regions — a pattern consistent with heightened stress reactivity and diminished capacity for top-down regulation of emotion. Importantly, these changes show evidence of partial reversal following structured intervention, suggesting that the damage is not irreversible if detected early (27). This partial reversibility is precisely what makes allostatic load a suitable target for condition-based monitoring: it is a progressive process with identifiable stages and a window of opportunity for intervention before changes become entrenched.

For monitoring purposes, allostatic load is typically indexed through a composite of routinely available biomarkers — cortisol, blood pressure, waist-hip ratio, HbA1c, and lipid panels — though as noted in our limitations, no standardised panel or scoring algorithm has been endorsed for psychiatric applications. This domain therefore represents the strongest conceptual case for condition-based monitoring but the least mature measurement infrastructure, and its clinical implementation awaits consensus on panel composition and prospective validation against psychiatric outcomes.

### 3.4 Autonomic function

Heart rate variability (HRV) — the beat-to-beat fluctuation in cardiac rhythm, reflecting the dynamic balance between sympathetic activation and parasympathetic (vagal) regulation — has emerged as a transdiagnostic biomarker of psychiatric vulnerability. A meta-analysis of 18 studies in patients with major depression without cardiovascular disease found reduced HRV across multiple indices, including high-frequency power (Hedges’ *g* = −0.29) and time-domain measures (*g* = −0.30) (28). A network meta-analysis of 42 studies (*N* = 4,008) confirmed that patients with major depression, generalised anxiety disorder, and panic disorder all exhibit significantly reduced HRV compared with healthy controls, with depression-specific reductions in the root mean square of the successive differences between normal heartbeats (RMSSD) (SMD = −0.64, 95% CI: −1.03 to −0.25) and the standard deviation of NN intervals (SDNN) (SMD = −0.40, 95% CI: −0.55 to −0.26) (29). Most recently, an umbrella review of 21 systematic reviews encompassing 442 primary studies and 34,625 participants synthesised HRV evidence across 19 mental disorders and found consistent patterns of reduced HRV, though the evidence for major depression specifically was classified as weak to suggestive rather than convincing (30). Importantly, the umbrella review found that no two disorders exhibited identical HRV alteration patterns, suggesting that HRV profiles — rather than individual HRV indices — may have diagnostic discriminative value.

These associations are clinically actionable because HRV is modifiable. A meta-analysis of 14 randomised controlled trials (*N* = 794) demonstrated that HRV biofeedback — a technique in which individuals learn to increase cardiac variability through paced breathing and real-time physiological feedback — significantly reduces depressive symptoms (Hedges’ *g* = 0.38, 95% CI: 0.16–0.60, *p* = .0006) (31). The physiological basis for this effect is well characterised: slow-paced breathing at approximately six cycles per minute approximates the resonance frequency of the cardiovascular system, maximising baroreflex gain and respiratory sinus arrhythmia, thereby stimulating the vagus nerve and enhancing parasympathetic regulation (32, 33). A Stanford randomised controlled trial in healthy volunteers reporting elevated subjective stress demonstrated that five minutes of daily cyclic sighing outperformed mindfulness meditation for mood improvement over 28 days (34), though generalisation to clinical populations remains to be tested.

This domain also intersects with environmental exposure. Ambient air pollution contributes to psychiatric risk through systemic inflammation: inhaled particulate matter triggers peripheral immune activation and neuroinflammation, overlapping mechanistically with the immunological pathways discussed below. A UK Biobank study (*N* = 389,185) found that individuals in the highest quartile of air pollution exposure had a 16% increased hazard of developing depression (HR = 1.16) (35). Unlike the other domains, where intervention operates primarily at the individual clinical level, air pollution mitigation requires population-level policy measures — emissions regulation, urban green-space planning, and reduction of residential proximity to traffic — positioning it as a public health rather than clinical intervention target.

For condition-based monitoring, resting HRV (specifically RMSSD, a vagally mediated time-domain index) is continuously measurable through ambulatory devices and represents one of the few parameters in this framework that provides real-time physiological data between clinical contacts. Its value lies less in diagnostic specificity — the effect sizes for depression are modest and the evidence base is weaker than for sleep or inflammatory markers — than in its capacity to capture autonomic flexibility, a transdiagnostic indicator of the body’s ability to adapt to changing demands. Declining HRV may therefore function as an early, non-specific signal of physiological dysregulation that gains interpretive value when considered alongside concordant changes in other monitored domains.

### 3.5 Psychoneuroimmunological monitoring

A growing body of evidence indicates that depression is accompanied by a measurable shift in immune function. A comprehensive meta-analysis comparing 5,166 depressed patients with 5,083 controls found significantly elevated levels of several pro-inflammatory signalling molecules — including interleukin-6 (IL-6; Hedges’ *g* = 0.61), tumour necrosis factor alpha (TNF-α; *g* = 0.54), and C-reactive protein (CRP; *g* = 0.71) — alongside reduced levels of the anti-inflammatory marker IL-4 (36). A separate systematic review and meta-analysis (37 studies; 13,541 depressed patients, 155,728 controls) established the clinical prevalence of this phenomenon: based on 30 studies, approximately 27% of depressed patients show low-grade inflammation (CRP >3 mg/L), and 58% show mildly elevated levels (CRP >1 mg/L) (37). These prevalence estimates were not moderated by sample source, antidepressant treatment, age, BMI, or ethnicity.

The mechanistic relevance of these associations is well characterised. Miller and Raison’s (2016) (38) framework identifies five pathways by which inflammatory molecules produced in the body can cross into the brain, where they disrupt the synthesis of mood-regulating neurotransmitters (such as serotonin), dysregulate the hypothalamic–pituitary–adrenal (HPA) axis — the body’s central stress-response system — and impair the brain’s capacity for neuroplastic adaptation. In effect, peripheral inflammation reprogrammes the brain toward a state of heightened threat sensitivity and diminished recovery capacity. Importantly, inflammation in depression does not associate uniformly with all symptoms: pooled analyses from the UK Biobank and the NESDA cohort demonstrated that elevated CRP was most strongly associated with neurovegetative symptoms — appetite change (OR = 1.25), fatigue (OR = 1.12) — with weaker but still significant associations with sleep problems (OR = 1.05) and depressed mood (OR = 1.06), all of which persisted after adjustment for BMI (39). This differential pattern of association, in which energy-related symptoms show the strongest inflammatory signal, is consistent with the emerging concept of an immuno-metabolic depression subtype (40) and has direct implications for monitoring: elevated CRP in the context of prominent neurovegetative symptoms may represent a stronger clinical signal than isolated CRP elevation.

The causal relevance of the inflammatory pathway is supported by intervention evidence. A meta-analysis of anti-cytokine treatments — conducted primarily in populations with chronic inflammatory conditions such as rheumatoid arthritis and psoriasis rather than primary depression — found a clinically meaningful antidepressant effect (SMD = 0.40), particularly in patients with elevated baseline CRP (41). Critically, the antidepressant effect was associated with baseline depressive symptom severity but not with improvement in the primary physical illness, suggesting that mood improvement is at least partially independent of somatic recovery and consistent with a direct role for inflammatory pathways in depressive pathophysiology. Beyond identifying risk, CRP may also guide treatment selection: in the GENDEP trial, CRP differentially predicted response to serotonergic versus noradrenergic antidepressants, with the CRP × drug interaction accounting for 10.6% of individual-level variance in treatment outcome (42). This positions CRP as the most clinically actionable parameter in the proposed framework — not merely a risk indicator but a treatment-stratifying biomarker.

An important caveat is warranted, however: group-level associations do not translate straightforwardly to individual-level prediction. The medium effect sizes reflect substantial distributional overlap between depressed and non-depressed populations, and the fact that 73% of depressed patients do not show CRP >3 mg/L means that inflammatory markers alone will miss the majority of cases. Inflammatory markers are therefore best understood not as standalone diagnostic tests but as one component within a multi-domain monitoring system, contributing incremental risk information that gains predictive value when combined with concordant signals from other domains.

## 4 Discussion

The evidence synthesised in this review converges on a single proposition: psychiatry’s prevailing intervention model is miscalibrated. The field continues to operate at what might be termed a minimum-threshold specification — intervening at the point of established diagnosis — while the evidence now supports a substantially earlier, multi-domain, condition-based approach. The magnitude of the intervention threshold gap is not trivial: a 43% reduction in MDD incidence through pre-threshold intervention (10), a 70% greater likelihood of treatment response with shorter duration of untreated illness (11), and a 30% reduction in treatment discontinuation through early intervention services for first-episode psychosis (12) collectively represent a paradigm shift with major implications for service design, resource allocation, and clinical training.

The clinical staging framework (7, 8) provides the necessary diagnostic infrastructure for operationalising this shift. By defining graduated clinical states from at-risk (Stage 0) through attenuated syndromes (Stages 1a/1b) to established disorder (Stage 2+), staging enables clinicians to match intervention intensity and modality to the individual’s current position on the illness trajectory — precisely the logic of condition-based maintenance. The five domains reviewed here — sleep, nutrition, stress physiology, autonomic function, and immune signalling — represent modifiable parameters that could be integrated into routine staging assessments, creating a multi-dimensional monitoring protocol analogous to the condition-monitoring systems used in engineering. The Lancet Psychiatry Commission on youth mental health has recently called for precisely this kind of integrated, platform-based approach to early intervention (3), lending institutional weight to the framework proposed here.

Physical activity, which a recent network meta-analysis of 218 randomised controlled trials found to be comparable in efficacy to psychotherapy and antidepressants (43), was not included as a separate monitoring domain. Our rationale is conceptual rather than empirically demonstrated: exercise is known to improve sleep architecture, reduce inflammatory markers, lower allostatic load, and enhance vagal tone, which suggests that its psychiatric effects may be substantially mediated through the pathways already represented in this framework. However, we acknowledge that this mediational claim has not been formally tested — no study has demonstrated that controlling for improvements in these five domains eliminates the independent antidepressant effect of exercise — and that physical activity likely also exerts effects through mechanisms not captured by the framework, including BDNF-mediated hippocampal neuroplasticity and direct monoaminergic modulation. Furthermore, physical activity could equally be conceptualised as a parsimonious cross-domain proxy: a single behaviour whose deterioration signals degradation across multiple monitoring parameters simultaneously. The exclusion of physical activity as a separate domain is therefore a pragmatic design choice reflecting the framework’s emphasis on measurable biological parameters rather than behaviours, not an evidence-based claim that its effects are fully accounted for by the included domains.

### 4.1 Monitoring protocol

**Table 1** presents a speculative monitoring protocol illustrating how the five domains could be operationalised within a condition-based framework. Three design principles warrant explanation.

First, monitoring frequency is calibrated both to the temporal dynamics of each domain and to clinical stage. Sleep architecture and autonomic function fluctuate over days to weeks and are suited to continuous ambulatory monitoring with periodic clinical review, whereas inflammatory and allostatic load markers change over months and require periodic blood sampling — analogous to the distinction between real-time sensors and scheduled inspections in engineering. Monitoring intervals shorten as staging classification advances: at Stage 0, 6-monthly biomarker assessment may suffice, whereas Stage 1a or above warrants quarterly review.

Second, action thresholds are defined relative to individual baselines rather than population norms where possible, reflecting the condition-based maintenance principle that deterioration from a known state is more informative than deviation from a population mean.

Third, the immune domain warrants comment. CRP thresholds are among the most clinically mature parameters in this framework: approximately 27% of depressed patients show low-grade inflammation (CRP >3 mg/L), and over half show mildly elevated levels (>1 mg/L) (37). Importantly, CRP does not merely identify risk — it can inform treatment selection: in the GENDEP trial, CRP differentially predicted response to serotonergic versus noradrenergic antidepressants, with the CRP × drug interaction accounting for 10.6% of individual-level variance in outcome (42). Furthermore, inflammation in depression associates most strongly with energy-related neurovegetative symptoms — appetite change, fatigue, and hypersomnia — rather than uniformly across the symptom spectrum (39), consistent with the emerging immuno-metabolic depression subtype (40). This symptom-specificity has direct implications for monitoring: elevated CRP in the context of energy-related symptoms may represent a stronger signal for intervention than isolated CRP elevation.

### 4.2 Discordant signals

When monitoring parameters diverge — for example, when inflammatory markers are elevated but sleep architecture remains intact, or when allostatic load indices rise without corresponding mood changes — the staging framework provides interpretive guidance. Single-domain anomalies at Stage 0 may warrant targeted domain-specific intervention (e.g., dietary modification for isolated inflammatory elevation) without necessitating stage reclassification. Conversely, concordant deterioration across multiple domains — simultaneous sleep disruption, rising CRP, and declining heart rate variability — would constitute a stronger signal for stage transition and broader intervention. This multi-parameter, convergent-evidence approach mirrors the logic of condition-based maintenance in complex systems, where single-parameter anomalies trigger heightened monitoring while multi-parameter convergence triggers intervention.

We emphasise that this protocol is speculative: none of the proposed thresholds have been validated in prospective multi-domain monitoring trials, and the protocol is presented to demonstrate conceptual feasibility and to identify research priorities for empirical testing.

### 4.3 Limitations

Several limitations require acknowledgement. First, the evidence base, while robust in several domains, remains incomplete. The Buntrock et al. (2024) (10) meta-analysis showed that preventive effects attenuated by 24 months, suggesting that optimal maintenance protocols — including re-intervention schedules — remain to be defined.

Second, effect sizes vary considerably across contexts: the pooled dietary improvement effect on depression is modest in predominantly non-clinical samples (*g* = 0.162) (21), while individual trials in clinically depressed populations show substantially larger effects (*d* = 1.16) (18), underscoring the importance of matching intervention intensity to stage of illness.

Third, cost-effectiveness data for integrated preventive programmes are encouraging but geographically limited, and implementation science research will be essential for translating these findings into routine clinical practice.

Fourth, the allostatic load construct, while conceptually central to this framework, faces a significant measurement challenge. Researchers have operationalised allostatic load using anywhere from 6 to over 20 biomarkers spanning neuroendocrine, cardiovascular, metabolic, and immunological systems, with no consensus on which markers should be included, how they should be weighted, or what scoring algorithm should be applied (24). Associations with psychiatric outcomes vary depending on panel composition — complicating direct comparison across studies and undermining the reliability of any single threshold as a clinical action point. The Gou et al. (2025) (25) findings cited in this review used a 10-biomarker panel, but different panel compositions would likely yield different risk estimates. This contrasts with CRP, which has emerged as a single, standardised marker with established risk thresholds (37). Until the field converges on a standardised allostatic load panel validated against psychiatric outcomes, this domain remains the least measurement-ready of the five proposed.

Fifth, the engineering analogy, although conceptually clarifying, carries important limitations that must be explicitly acknowledged. Human beings differ from engineered systems in at least three fundamental respects. Biological systems are self-repairing: unlike a mechanical component that degrades monotonically until replacement, the brain exhibits neuroplasticity, immune recalibration, and HPA axis re-regulation — capacities that mean deterioration detected at an early stage may resolve spontaneously or with minimal intervention, a possibility with no direct parallel in machinery maintenance. The partial reversibility of burnout-related brain changes (27) illustrates this capacity. Human health is also shaped by meaning, agency, and social context in ways that no engineering model captures. A patient’s interpretation of their symptoms, their sense of purpose, the quality of their relationships, and their socioeconomic circumstances all modulate illness trajectories in ways that cannot be reduced to measurable biological parameters — and a purely parameter-driven monitoring framework risks obscuring these dimensions. Furthermore, the relationship between monitoring and the system being monitored is different: in engineering, measuring vibration frequency does not change the machine, whereas in psychiatry, the act of monitoring itself — being screened, receiving biomarker results, being assigned a clinical stage — may alter the patient’s psychological state, for better or worse. These disanalogies do not invalidate the framework, but they constrain its interpretation. We intend the condition-based maintenance analogy as a heuristic for reorienting clinical thinking from reactive to preventive — not as a claim that psychiatric care can or should be reduced to parameter-driven algorithmic decision-making. The framework identifies what to monitor and when to intervene; the clinical judgement of how to intervene, and the therapeutic relationship within which intervention occurs, remain irreducibly human.

Finally, any proposal for more frequent monitoring must contend with the well-documented potential harms of screening. The Canadian Task Force on Preventive Health Care recently issued a strong recommendation against routine depression screening using questionnaire-based cut-off scores, citing the absence of demonstrated benefit over usual care and the likelihood of false positives, overdiagnosis, and diversion of scarce resources (44). At a commonly used PHQ-9 cut-off of ≥10, screening 100 primary care patients yields approximately 13 false positives against 9 true positives (44). More broadly, overdiagnosis in mental health — whether through over detection of self-limiting conditions or over definition of diagnostic criteria that capture transient distress — remains poorly defined and unquantified (45). The condition-based framework proposed here differs from conventional questionnaire-based screening in that it emphasises biological parameters that may offer greater diagnostic specificity, and it incorporates individual baseline-referenced thresholds rather than population-level cut-offs — features that could, in principle, reduce false positive rates. However, these potential advantages are theoretical and untested. Any implementation trial should therefore include systematic assessment of screening-related harms as a primary outcome.

### 4.4 Implementation barriers

Beyond these evidential limitations, several practical barriers warrant acknowledgement. First, while the individual biomarkers that compose allostatic load indices are routinely collected in primary care and cardiometabolic medicine, they are not currently aggregated, scored, or interpreted as a composite psychiatric risk index in any health system. The infrastructure gap is less about the availability of individual measurements than about the absence of clinical workflows, automated scoring algorithms, and electronic health record integrations that would transform existing data into actionable psychiatric risk information. This stands in contrast to CRP, which is a single, inexpensive, widely available blood test with established clinical thresholds (37) and demonstrated treatment-selection utility (42), and which is therefore substantially closer to clinical implementation.

Second, the proposed multi-domain monitoring cannot be accommodated within the 10–15-minute primary care consultations that characterise most health systems. Realistic implementation would likely require dedicated preventive mental health appointments — analogous to the structured cardiovascular risk assessments that many countries have successfully integrated into primary care — or delegation of routine monitoring to allied health professionals, digital platforms, or both.

Third, the emphasis on ambulatory monitoring technologies raises legitimate equity concerns. Digital health innovations risk exacerbating rather than reducing health inequalities — a phenomenon termed the “digital health paradox” (46). Access to personal monitoring devices, reliable internet connectivity, and digital health literacy is unevenly distributed across socioeconomic strata and geographic regions — precisely the populations that often bear the highest burden of mental illness. Any implementation strategy must therefore include low-technology alternatives to avoid creating a two-tier preventive system that deepens existing health inequities.

Fourth, the clinical workforce is not currently trained for this model. Implementing condition-based care would require substantial curricular reform in psychiatric training, including familiarity with inflammatory markers, allostatic load indices, heart rate variability interpretation, and the principles of clinical staging. These barriers are substantial but not unprecedented: the integration of cardiovascular risk assessment into primary care required analogous infrastructure development, workforce retraining, and institutional commitment over a period of decades.

### 4.5 Future research priorities

Three priorities emerge. The most urgent is the design and execution of prospective multi-domain monitoring trials that test whether the integrated framework proposed here — rather than any single domain in isolation — improves psychiatric outcomes relative to current practice. Such trials should employ adaptive designs that can evaluate different monitoring intervals, action thresholds, and domain combinations while including systematic assessment of screening-related harms.

Second, health-economic modelling is needed to determine whether the upfront costs of multi-domain monitoring are offset by reduced downstream treatment costs, disability, and lost productivity — an analysis that will be essential for policy adoption.

Third, the development and validation of composite biomarker panels — particularly for allostatic load — and their integration with ambulatory physiological monitoring represents a critical translational step. Advances in computational approaches to behavioural and physiological data may eventually enable ongoing condition assessment between clinical contacts (47), but these technologies must be validated against psychiatric outcomes and implemented in ways that ensure equitable access.

The convergence of clinical staging, multi-domain biological monitoring, and advances in ambulatory assessment offers a realistic path toward a preventive psychiatry that intervenes at the right time, with the right tools, across the right domains. Whether the field will take this path depends not on the availability of evidence — which, as this review demonstrates, is already substantial — but on the willingness to restructure services, retrain clinicians, and invest in prevention rather than continuing to wait for the system to fail.
